# A nomogram for predicting the recurrence of small bowel obstruction after gastrectomy in patients with gastric cancer

**DOI:** 10.1186/s12957-023-03197-1

**Published:** 2023-11-09

**Authors:** Wenhao Yu, Qi Zhang, Muhammad Ali, Bangquan Chen, Yapeng Yang, Liuhua Wang, Qiannan Sun, Yong Wang, Daorong Wang

**Affiliations:** 1 Department of Gastrointestinal Surgery, Northern Jiangsu People’s Hospital Affiliated to Yangzhou University, No.98 Nantong West Road, Yangzhou, Jiangsu Province China; 2 General Surgery Institute of Yangzhou, Yangzhou University, Yangzhou, China; 3 Medical College of Yangzhou University, Yangzhou, China

**Keywords:** Gastric cancer, Small bowel obstruction, Recurrence, Risk factors, Nomogram

## Abstract

**Background:**

This study aimed to create a nomogram for predicting the recurrence of small bowel obstruction (SBO) after gastrectomy in patients with gastric cancer (GC) in order to provide better guidance for its diagnosis and treatment.

**Methods:**

A total of 173 patients undergone gastrectomy and developed SBO from January 2015 to October 2022 were admitted into this case–control study. The risk factors of postoperative recurrent SBO were analyzed by univariate and multivariate regression, and a nomogram for predicting the recurrent SBO after gastrectomy was developed using R Studio.

**Results:**

Thirty-nine cases of postoperative recurrent SBO occurred among the 173 GC patients who underwent radical gastrectomy, and the percentage of recurrent SBO was 22.54% (39/173). Age [odds ratio (OR) = 0.938, *p* = 0.026], WBC count (OR = 1.547, *p* < 0.001), tumor size (OR = 1.383, *p* = 0.024), postoperative metastasis (OR = 11.792, *p* = 0.030), and the interval from gastrectomy to first SBO (OR = 1.057, *p* < 0.001) were all identified as independent risk factors for postoperative recurrent SBO by logistic regression analysis. The receiver operating characteristic curve, the calibration curve, the model consistency index, and the decision curve analysis showed that the nomogram had good predictive performance.

**Conclusion:**

Based on these factors, we created a nomogram to predict the occurrence of postoperative recurrent SBO. This novel nomogram could serve as a crucial early warning indicator that would guide doctors to make informed decisions while managing patients with gastric cancer.

## Background

Gastric cancer (GC) is the fifth most common cancer worldwide and the third leading cause of cancer-related deaths [1]. Currently, gastrectomy combined with lymph node dissection remains the main treatment modality for GC [2]. However, patients who undergo surgical treatment for GC are at an increased risk of developing postoperative small bowel obstruction (SBO) owing to the numerous procedures that are performed, such as reconstruction of the digestive tract, lymphatic dissection, and severing of the omentum majus and mesentery. A previous study reported that nearly 93% of patients who had previously undergone one or more abdominal surgeries developed intra-abdominal organ adhesions or bowel adhesions [3], which may originate from tissue trauma, inflammatory responses, and the healing process.

SBO is a condition that causes mechanical obstruction that interrupts the flow of intestinal contents, resulting in proximal bowel dilation and distal bowel decompression [2]. Constipation, nausea, vomiting, and abdominal discomfort are all clinical manifestations of SBO. SBO is one of the common causes of emergency hospital admissions worldwide, accounting for approximately 17% of surgical emergency admissions [4], so it requires prompt treatment after admission to prevent intestinal necrosis and potentially life-threatening complications. Moreover, postoperative SBO poses a significant obstacle to the recovery of these patients who have undergone gastrectomy, which greatly reduces their quality of life and increases the cost burden of treatment, and recurrent SBO adds further stress to the patient’s life.

Most of the previous studies on postoperative SBO have only focused on the effect of the different surgical methods, but few studies have investigated the risk factors for postoperative recurrent SBO. Some scholars have studied the effect of digestive tract reconstruction on postoperative SBO, and they believe that retrocolic anastomosis can significantly reduce the incidence of postoperative SBO [5]. Another study also studied the influence of surgical methods, and the results proved that total gastrectomy and greater omentectomy were significant risk factors for postoperative SBO [6]. To further investigate the risk factors of postoperative recurrent SBO, we conducted this study and aimed to create a prediction nomogram to assess the probability of recurrent SBO after gastrectomy for GC in the study future. This nomogram could help doctors to increase the frequency of follow-up for patients who are prone to SBO. Early interventions could be implemented to prevent disease progression if any signs or symptoms of SBO arise.

## Patients and methods

### Study subjects

The historical records of 2218 patients who had undergone radical surgery for GC at the General Surgery Department of Northern Jiangsu People’s Hospital from January 2015 to October 2020 were collected. This study was performed in line with the principles of the Declaration of Helsinki. The study protocol was approved by the Ethics Committee of the Northern Jiangsu People’s Hospital, Clinical Medical School, Yangzhou University (approval no. 2019KY-022). The inclusion criteria for the study were adult patients with pathologically proven gastric cancer of any stage who underwent radical gastrectomy and recovered well from surgery and developed at least one episode of SBO. The exclusion criteria were patients who did not experience postoperative intestinal obstruction; those with other serious co-morbidities, such as other malignancies, severe hepatic or renal dysfunction, or heart failure; those with incomplete clinical, laboratory, or surgical data or data that could not be analyzed; and those who died within 30 days after GC surgery or had gastric stump carcinoma.

### Variables and definitions

A total of 173 patients were enrolled in this study. These patients were divided into two groups according to whether there was a recurrence of SBO: the recurrence group and the control group consisted of patients without recurrence. Through the hospital’s electronic medical records, the following information was collected for each patient: age, gender, previous abdominal surgery, co-morbidities (diabetes, hypertension, and heart disease), history of smoking and drinking, body mass index (BMI), hematological index before gastrectomy [CA199 (carbohydrate antigen199), AFP (alpha fetoprotein), CEA (carcinoembryonic antigen), WBC (white blood cell) count, and albumin level], time of GC surgery, intraoperative blood transfusion, intraoperative bleeding, surgical approach, tumor location, tumor size, surgical procedure, reconstruction method, combined organ resection, close Petersen’s defect, total length of stay, postoperative hospital stay, postoperative metastasis, pathological TNM stage, pathological type, invasion of nerve, transfer of vessel, abdominal pain, abdominal distension, nausea, vomiting, stop farting, cessation of bowel movements, treatment of SBO, fart time after treatment, defecation time after treatment, and the interval from gastrectomy to first SBO (which refers to the time between the completion of gastrectomy and the first onset of SBO) were gathered.

SBO is confirmed based on the presence of clinical symptoms (nausea, vomiting, abdominal pain, abdominal distension, and the absence of defecation or flatus within the previous 24 h) and the results of physical examinations (abdominal tenderness, accentuation of bowel sounds, tympanitic sounds on percussion), plain abdominal radiographs (an air-fluid level in the upright position), and enhanced computed tomography (CT) scans (dilatation of the small intestine). SBO is objectively diagnosed according to findings observed on plain abdominal radiographs or enhanced CT scans, as well as the presence of at least three of the aforementioned clinical symptoms and the results of a physical examination. These diagnostic criteria have been used in several studies [7, 8]. The occurrence of two or more SBOs after gastrectomy is considered to be postoperative recurrent SBO.

### Statistical analysis

Software such as SPSS 25.0 and R Studio with R packages of rms, reader, ResourceSelection, rmda, pROC, and nomogramEx were utilized for statistical analysis. Normally distributed data were compared using the mean (± standard deviation) and the *t* test, whereas non-normally distributed data were compared using the median (interquartile range) and the Mann–Whitney *U* test. Categorical data were compared using the chi-square test. Statistical significance was determined using a 95% confidence interval, with a *p* value < 0.05 indicating a significant difference. To investigate the risk factors of postoperative recurrent SBO, univariate and multivariate logistic regression analysis was applied to investigate the interaction of variables. Because nomograms have the primary advantage of the ability to estimate individualized risk based on patient and disease characteristics [9], we created a nomogram to determine the association between GC surgery and the recurrence of SBO based on the results of the multivariate analysis. Harrell’s concordance index (C-index) was used to assess the predictive ability of the nomogram. Internal validation of the nomogram was performed using 1000 bootstrap resamples to evaluate the difference between the actual and predicted probabilities. The prediction effectiveness of the generated nomogram was assessed using the receiver operating characteristic (ROC) curve. The clinical usefulness of the nomogram was assessed using decision curve analysis (DCA).

## Results

### Baseline characteristics

After excluding patients who did not develop SBO after gastrectomy, a total of 173 patients with postoperative SBO were included in this study. The overall incidence of postoperative SBO was 7.8% (173/2218). Among the 173 patients with SBO, 39 had recurrent SBO and 134 had SBO without recurrence, with a recurrence rate of 22.54% (39/173). In the recurrence group, the median age was 62 (range 58–69 n the rethe median WBC count was 7.0 (5.2, 10.5 × 10^9^/L), the median tumor size was 6 (5, 6 cm), the postoperative metastasis rate was 15.4% (6/39), and the median interval from gastrectomy to first SBO was 34 (23–43 months). In the non-recurrence group, the median age was 68 (60–72), the median WBC count was 5.7 (4.5, 6.9), the median tumor size was 5 (3, 6), the postoperative metastasis rate was 1.5% (2/134), and the median interval from gastrectomy to first SBO was 9 (5–18).

### Univariate analysis results

The results of univariate analysis indicated that there were no significant differences in terms of gender, history of previous abdominal surgery, co-morbidities (hypertension, diabetes, heart disease), history of smoking and drinking, BMI, requirement of intraoperative blood transfusion, intraoperative bleeding, surgical approach, and tumor location between the two groups (*p* > 0.05). Statistically significant differences were observed in factors such as age, CEA, WBC count, albumin level, time of GC surgery, tumor size, postoperative metastasis, nerve invasion, and the interval from gastrectomy to first SBO (*p* < 0.05). Detailed information is provided in Table 1.

**Table 1:** Comparison of the general conditions of patients in the two groups

Factors	Recurrence	Non-recurrence	*χ*^2^/*t*	*p* value
Gender			1.286	0.257
Men	32 (82.1%)	98 (73.1%)		
Women	7 (17.9%)	36 (26.9%)		
Age (years)	62 [58,69]	68 [60,72]		0.015*
Previous abdominal surgery			0.261	0.610
Yes	10 (25.6%)	40 (29.9%)		
No	29 (74.4%)	94 (70.1%)		
Diabetes			0.152	0.697
Yes	2 (5.1%)	5 (3.7%)		
No	37 (94.9%)	129 (96.3%)		
Hypertension			1.553	0.213
Yes	9 (23.1%)	45 (33.6%)		
No	30 (76.9%)	89 (66.4%)		
Heart disease			2.608	0.106
Yes	4 (10.3%)	5 (3.7%)		
No	35 (89.7%)	129 (96.3%)		
Smoking			0.230	0.632
Yes	12 (30.8%)	36 (26.9%)		
No	27 (69.2%)	65 (48.5%)		
Drinking			0.010	0.920
Yes	7 (17.9%)	25 (18.7%)		
No	32 (82.1%)	109 (81.3%)		
BMI (kg/m^2^)	22.4 (± ± .4)	23.1 (± ± .1)		0.234
CA199 (ng/mL)	10.2 [6.3, 18.7]	8.4 [5.0, 11.2]		0.096
AFP (ng/mL)	2.09 [1.5, 3.3]	1.7 [1.3, 2.8]		0.113
CEA (ng/mL)	2.7 [1.7, 4.5]	1.8 [1.3, 2.9]		0.013*
WBC count (× 10^9^/L)	7.0 [5.2, 10.5]	5.7 [4.5, 6.9]		< 0.001*
Albumin level (g/L)	38.1 [35.2, 43.4]	42.2 [36.7, 46.2]		0.033*
Time of GC surgery (min)	180 [150, 190]	160 [132, 180]		0.021*
Intraoperative blood transfusion			0.204	0.652
Yes	1 (2.6%)	2 (1.5%)		
No	38 (97.4%)	132 (98.5%)		
Intraoperative bleeding (ml)	100 [50, 200]	200 [100, 200]		0.705
Surgical approach			1.487	0.223
Laparoscopic	16 (41.0%)	41 (30.6%)		
Open	23 (59.0%)	93 (69.4%)		
Tumor location			0.462	0.497
Cardia/Fundus	16 (41.0%)	47 (35.1%)		
Gastric body/Pylorus	23 (59.0%)	87 (64.9%)		
Tumor size (cm)	6 [5, 6]	5 [3, 6]		0.011*
Surgical procedure			0.639	0.726
Total gastrectomy	33 (84.6%)	107 (79.9%)		
Proximal gastrectomy	1 (2.6%)	7 (5.2%)		
Distal gastrectomy	5 (12.8%)	20 (14.9%)		
Reconstruction method			3.561	0.313
Roux-en-Y	35 (89.7%)	122 (91.0%)		
Uncut Roux-en-Y	3 (7.7%)	3 (2.2%)		
Billroth-1	0 (0%)	1 (0.7%)		
Billroth-2	1 (2.6%)	8 (6.0%)		
Combined organ resection			0.889	0.346
Yes	0 (0%)	3 (2.2%)		
No	39 (100%)	131 (97.8%)		
Close Petersen’s defect			0.336	0.562
Yes	32 (82.1%)	115 (85.8%)		
No	7 (17.9%)	19 (14.2%)		
Total length of stay (days)	15 [14, 19]	16 [14, 19]		0.337
Postoperative hospital stay (days)	13 [12, 13]	13 [12, 14]		0.091
Postoperative metastasis			13.218	< 0.001*
Yes	6 (15.4%)	2 (1.5%)		
No	33 (84.6%)	132 (98.5%)		
Pathological *T* stage			3.407	0.333
T1	5 (12.8%)	32 ( 23.9%)		
T2	6 (15.4%)	11 (8.2%)		
T3	5 (12.8%)	15 (11.2%)		
T4	23 (59.0%)	76 (56.7%)		
Pathological *N* stage			1.074	0.753
N0	15 (38.5%)	53 (39.6%)		
N1	5 (12.8%)	25 (18.7%)		
N2	8 (20.5%)	21 (15.7%)		
N3	11 (28.2%)	35 (26.1%)		
Pathological TNM stage			2.368	0.306
I	17.9 (1%)	38 (28.4%)		
II	11 (28.2%)	26 (19.4%)		
III	21 (53.8%)	70 (52.2%)		
Pathological type			1.416	0.702
Adenocarcinoma	37 (94.9%)	109 (81.3%)		
Mucinous adenocarcinoma	1 (2.6%)	2 (1.5%)		
Signet-ring cell carcinoma	1 (2.6%)	1 (4.5%)		
Neuroendocrine carcinoma	0 (5.1%)	2 (0.7%)		
Invasion of nerve			5.735	0.017*
Yes	22 (56.4%)	47 (35.1%)		
No	17 (43.6%)	87 (64.9%)		
Transfer of vessel			1.581	0.209
Yes	18 (46.2%)	47 (35.1%)		
No	21 (53.8%)	87 (64.9%)		
Abdominal pain			3.419	0.064
Yes	39 (100%)	123 (91.8%)		
No	0 (0%)	11 (8.2%)		
Abdominal distension			0.072	0.788
Yes	25 (64.1%)	89 (66.4%)		
No	14 (35.9%)	45 (33.6%)		
Nausea			0.784	0.376
Yes	26 (66.7%)	99 (73.9%)		
No	13 (33.3%)	35 (26.1%)		
Vomiting			1.560	0.212
Yes	18 (46.2%)	77 (57.5%)		
No	21 (53.8%)	57 (42.5%)		
Stop farting			0.245	0.620
Yes	25 (64.1%)	80 (59.7%)		
No	14 (35.9%)	54 (40.3%)		
Cessation of bowel movements			0.040	0.841
Yes	26 (66.7%)	87 (64.9%)		
No	13 (33.3%)	47 (35.1%)		
Treatment of SBO			0.010	0.920
Surgery	7 (17.9%)	25 (18.7%)		
Conservative	32 (82.1%)	109 (81.3%)		
Fart time after treatment (days)	2 [0, 5]	2 [0, 3]		0.290
Defecation time after treatment (days)	3 [0, 5]	2 [0, 5]		0.601
The interval from gastrectomy to first SBO (months)	34 [23, 43]	9 [5, 18]		< 0.001*

*BMI* body mass index, *SBO* small bowel obstruction, *CA199* carbohydrate antigen199;, *AFP* alpha fetoprotein, *CEA* carcinoembryonic antigen, *WBC* white blood cell

^*^Significant risk factor (*p* value < 0.05) by chi-square test, *t* test, or Mann–Whitney *U* test

### Results of multivariate analysis

The factors with significant differences in univariate analysis were substituted into multivariate analysis. The results of the multivariate logistic regression analysis demonstrated the relationship between different factors and the recurrence of postoperative SBO (Table 2). The analysis revealed that age [odds ratio (OR) = 0.938, 95% confidence interval (95% CI) 0.887lysis re*p* = 0.026], WBC count (OR = 1.547, 95% CI 1.23226], WBC*p* < 0.001), tumor size (OR = 1.383, 95% CI 1.043–1.832, *p* = 0.024), postoperative metastasis (OR = 11.792, 95% CI 1.264–110.042, *p* = 0.030), and the interval from gastrectomy to first SBO (OR = 1.057, 95% CI 1.031–1.085, *p* < 0.001) were independent risk factors for the postoperative recurrent SBO.

**Table 2:** Multivariate logistic regression analysis of recurrent SBO after GC surgery

Factors	SE	Wald	OR (95%CI)	*p* value
Age (years)	0.029	4.974	0.938 (0.887–0.992)	0.026*
CEA (ng/mL)	0.048	0.235	0.977 (0.889–1.074)	0.628
WBC count (× 10^9^/L)	0.116	14.094	1.547 (1.232–1.942)	< 0.001*
Albumin level (g/L)	0.044	0.085	0.987 (0.905–1.077)	0.771
Time of GC surgery (min)	0.008	0.975	1.008 (0.992–1.024)	0.323
Tumor size (cm)	0.144	5.089	1.383 (1.043–1.832)	0.024*
Postoperative metastasis	1.140	4.688	11.792 (1.264–110.042)	0.030*
Invasion of nerve	0.530	2.705	2.392 (0.846–6.763)	0.100
The interval from gastrectomy to first SBO (months)	0.013	18.209	1.057 (1.031–1.085)	< 0.001*

*SE* standard error, *OR* odds ratio, *CI* confidence interval, *SBO* small bowel obstruction, *CEA* carcinoembryonic antigen, *WBC* white blood cell

*Significant risk factor (*p* < 0.05)

### Development of a nomogram to predict the risk of recurrent SBO after gastrectomy

**Fig. 1:**
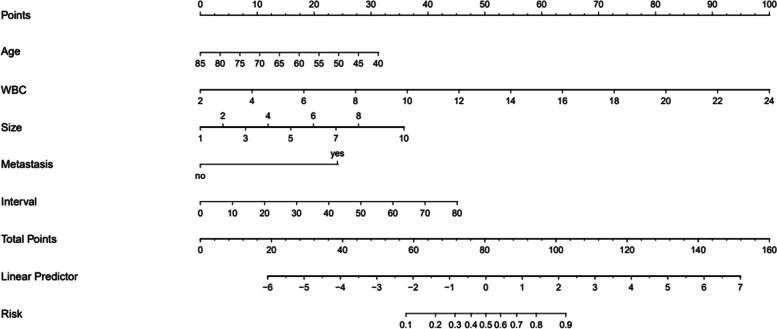
Nomogram predicting the probability of recurrent SBO after GC surgery. For an individual patient, points for each of the 5 risk factors are summed to give a total point. The horizontal axis representing the total points is then used to calculate the corresponding probability of recurrent SBO after GC surgery

The results of the multivariate logistic regression analysis were used to generate a nomogram for predicting the recurrence of SBO after gastrectomy (Fig. 1). The nomogram assigns scores to five independent risk factors: age, WBC count, tumor size, postoperative metastasis, and the interval from gastrectomy to first SBO. These scores are added to obtain a total score, which corresponds to different prediction probabilities of the postoperative recurrent SBO.

**Fig. 2:**
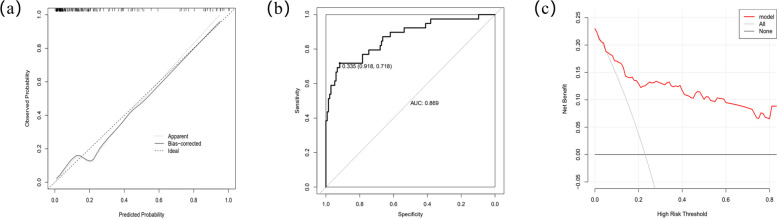
**a** Calibration plot of nomogram. The dashed line indicates the ideal nomogram in which predicted and actual probabilities were perfectly identical. The dotted line indicates actual nomogram performance. The solid line presents the bootstrap corrected performance of our nomogram (*B* = 1000 repetitions). The calibration plot illustrates good predictive accuracy. **b** The receiver operating characteristic (ROC) curve of the multivariate logistic regression model. **c** Decision curve analysis (DCA) for the nomogram. The *y*-axis represents the net benefit. The *x*-axis shows the threshold probability. “All” refers to the assumption that all patients develop recurrent SBO after gastrectomy and “None” to the assumption that no patient develops recurrent SBO

The performance of the generated nomogram was assessed using various evaluation metrics. The model C-index was 0.869 (95% CI 0.799–0.940), indicating that the nomogram had good accuracy in estimating the probability of recurrent SBO. The calibration plot with bootstrap sampling (*n* = 1000) summarized the performance characteristics of the nomogram (Fig. 2a). The bias-corrected curve was close to the ideal curve, indicating that the nomogram was well established and showed good calibration. The performance of this nomogram was measured using ROC curve analysis, and the area under the ROC curve (AUC) of this model was 0.869, indicating a good diagnostic performance with a sensitivity of 0.918 and a specificity of 0.718 at the optimal cutoff value (Fig. 2b). The DCA revealed a high net benefit which confirming that the nomogram had good clinical applicability in predicting the recurrence of postoperative SBO for GC (Fig. 2c).

## Discussion

Scholars only studied the risk factors of SBO after gastrectomy previously and did not study the effects of different factors on the recurrence of postoperative SBO. A randomized study studied the influence of the choice of incision mode during gastrectomy on postoperative complications, and the results showed that transverse incision may be more beneficial than middle and upper incision to reduce postoperative wound pain and the incidence of postoperative pneumonia and postoperative intestinal obstruction [10]. Another study identified previous abdominal surgery, open gastrectomy, non-Billroth-1 reconstruction, etc., are risk factors related to adhesive small bowel obstruction (ASBO) after gastrectomy, and based on these factors, a nomogram can be generated to predict the probability of ASBO [11]. Unlike previous studies, this study is the first to focus on the risk factors for recurrence of SBO after gastrectomy, using identified independent risk factors to create a nomogram to predict the probability of recurrence of SBO after gastric cancer surgery. In clinical work, we often encounter patients who are admitted to the hospital due to recurrent SBO after gastrectomy, some patients can be relieved with conservative treatment, and others need to be treated with surgery. However, patients still have a high probability of recurrent SBO after discharge, which brings huge psychological pressure and life burden to patients. Therefore, we believe that preventing the recurrence of SBO to avoid multiple hospitalizations is of great importance to both doctors and patients.

Few clinical studies have explored the effect of age and WBC count on recurrent SBO after surgery. Only a quicker peritoneal regeneration in the immature rat has been experimentally described [12]. A higher frequency of postoperative SBO after abdominal surgery in early life was reported, especially in the neonatal period [13]. In addition, gastrointestinal tract mobility decreases with age, which may have an impact on the recurrence of postoperative SBO [14]. Likewise, experimental studies have investigated the mechanism of leukocytes for postoperative SBO. Researchers proved that iNOS expressed in leukocytes plays a major role in mediating smooth muscle dysfunction and postoperative SBO [15]. Another experiment found that leukocyte-derived interleukin (IL)-10 could induce neutrophil extravasation into the postsurgical bowel wall, which increased the occurrence of postoperative SBO [16]. We included age and WBC count as risk factors in our study because they reflect the underlying physical condition of patients, and our findings showed that younger age and higher WBC count increased the risk of postoperative recurrent SBO.

The tumor size which was reflected by the tumor’s largest diameter was the first independent risk factor. In this study, there was a significant difference in tumor size between the two groups (*p* = 0.024). The study has proved that the larger the tumor size, the higher the risk of postoperative recurrent SBO in patients. Recently, Lv et al. [17] conducted a large retrospective cohort study of patients’ data in the Surveillance, Epidemiology, and End Results and Medicare claims-linked databases (SEER-M database), and they reported that tumor size was significantly associated with SBO according to the results of multivariate analysis. In addition, there was a study of complications after gastrectomy [18], and the results showed that tumor size was an independent prognostic factor for grade I complications after gastrectomy (*p* = 0.031). Therefore, we thought that tumor size may be related to postoperative recurrent SBO, which was confirmed in our study.

Postoperative metastases, such as peritoneal metastasis and ovarian metastasis, in patients with GC can significantly increase the risk of developing postoperative recurrent SBO. Metastases can increase pressure on the intestines, leading to intestinal strictures or direct blockage of the intestine, resulting in SBO. A study consisting of 61 patients who underwent surgical treatment following GC metastasis has reported malignant intestinal obstruction as the most common complication in these patients [19]. A Japanese study [20] identified lymph node metastasis and peritoneal dissemination as significant risk factors associated with obstructive tumors, indicating that tumor metastasis can influence the development of SBO. Other studies [21–23] have found that tumor metastasis from other sites to the gastrointestinal tract leads to SBO, such as large cell carcinoma of the lung, breast cancer, and renal cell carcinoma, which indirectly proves the correlation between postoperative metastasis of GC and SBO. The specific mechanism of metastasis leading to SBO requires us to conduct in-depth research in the future.

Finally, the results of multivariate regression analysis showed that OR = 1.057 (95% CI 1.031–1.085) for the interval from gastrectomy to first SBO, although the OR value was small, which may be due to the large difference in sample size between the two groups and the mutual influence of multiple factors when analyzed together [24]. A significant difference at *p* < 0.001 indicated that this variable was a risk factor for postoperative recurrent SBO, so it was included in our nomogram. One study [25] found no significant effect of elapsed time from the latest operation on adhesive postoperative SBO. However, some studies [26, 27] have shown that the risk of recurrence of SBO increases with time and the number of hospital admissions. Moreover, studies by Colonna et al. [28] have proved that the recurrence rate of SBO varies with time interval and the number of previous ileus occurrences, and the longer the time interval or with the increase in the number of ileus recurrence, the risk of recurrent SBO also increases. We hypothesized that the reason is that the longer the time, the worse the physical condition of the patients, and the more likely to suffer from malignant complications such as postoperative tumor metastasis, so the more likely to have multiple episodes of SBO. Currently, there are few studies on this variable, and more and more prospective studies should be conducted to examine this topic in the future.

We can explain the nomogram by the following steps: First, determine the value of the variable on the corresponding axis; second, draw a vertical line to the total points axis to determine the points; third, add the points of each variable; and finally, draw a line from the total point axis to determine the postoperative recurrent SBO probabilities at the lower line of the nomogram. For example, the age of patients with GC was 60 years old (17 points), the WBC count was 8 × 10^9^/L (27.5 points), the tumor size was 4 cm (12 points), no postoperative metastasis (0 points), and the interval from gastrectomy to first SBO was 20 months (11 points). Therefore, a total score of 67.5 corresponds to a recurrence probability of postoperative SBO of about 23%.

To the best of our knowledge, this study is the first to develop a nomogram to predict postoperative recurrent SBO. This novel nomogram may serve as a crucial early warning signal to identify patients at a higher risk of developing recurrent SBO after gastrectomy. Unfortunately, there is no way to completely prevent SBO currently. However, doctors should use any means possible to reduce the risk of recurrent SBO. We can use starch-free gloves during surgical procedures [29], irrigate the abdominal cavity with saline below 37 °C [30], preserve the omentum [6], and intraoperative application of preventive materials such as the antiadhesive agent [31], icodextrin 4% solution (Adept) [32], and hyaluronic acid/carboxymethylcellulose (Seprafilm) [33, 34] may reduce the recurrence rate of SBO in GC patients undergoing gastrectomy. Of course, we can also use conservative treatment methods, such as a manual physiotherapy called the Clear Passage Approach (CPA) [35], advising patients to have a liquid diet and increase exercise.

However, the study also has some limitations that should be acknowledged. First, the retrospective nature of the study and the limited sample size may have undermined the findings, and some uncontrolled confounders might also arise. In addition, further external validation in a multicenter setting is required to determine whether this nomogram could be widely used in other populations. Finally, there was no reliable independent cohort for validating the predictive efficiency of the nomogram. Despite these limitations, the study still has certain research value and provides valuable insights and references for future research on the risk assessment of postoperative recurrent SBO in patients with GC.

## Conclusion

In conclusion, our study found that age, WBC count, tumor size, postoperative metastasis, and the interval from gastrectomy to first SBO may be risk factors for the development of recurrent SBO after gastrectomy. We proposed a nomogram to predict the recurrence of SBO after gastrectomy in patients with GC. This novel nomogram may serve as an essential tool to assist doctors in estimating the risk of individual patients and implementing appropriate preventive measures. However, this study was a single-center retrospective cohort study, and the model has not been externally validated.

## Data Availability

The data of the article can be accessed by requesting the corresponding author.
